# genomepy: genes and genomes at your fingertips

**DOI:** 10.1093/bioinformatics/btad119

**Published:** 2023-03-06

**Authors:** Siebren Frölich, Maarten van der Sande, Tilman Schäfers, Simon J van Heeringen

**Affiliations:** Radboud University, Department of Molecular Developmental Biology, Faculty of Science, Radboud Institute for Molecular Life Sciences, Nijmegen 6525GA, The Netherlands; Radboud University, Department of Molecular Developmental Biology, Faculty of Science, Radboud Institute for Molecular Life Sciences, Nijmegen 6525GA, The Netherlands; Radboud University, Department of Molecular Developmental Biology, Faculty of Science, Radboud Institute for Molecular Life Sciences, Nijmegen 6525GA, The Netherlands; Radboud University, Department of Molecular Developmental Biology, Faculty of Science, Radboud Institute for Molecular Life Sciences, Nijmegen 6525GA, The Netherlands

## Abstract

**Motivation:**

Analyzing a functional genomics experiment, such as ATAC-, ChIP-, or RNA-sequencing, requires genomic resources such as a reference genome assembly and gene annotation. These data can generally be retrieved from different organizations and in different versions. Most bioinformatic workflows require the user to supply this genomic data manually, which can be a tedious and error-prone process.

**Results:**

Here, we present genomepy, which can search, download, and preprocess the right genomic data for your analysis. Genomepy can search genomic data on NCBI, Ensembl, UCSC, and GENCODE, and inspect available gene annotations to enable an informed decision. The selected genome and gene annotation can be downloaded and preprocessed with sensible, yet controllable, defaults. Additional supporting data can be automatically generated or downloaded, such as aligner indexes, genome metadata, and blacklists.

**Availability and implementation:**

Genomepy is freely available at https://github.com/vanheeringen-lab/genomepy under the MIT license and can be installed through pip or Bioconda.

## 1 Introduction

Data analysis is increasingly important in biological research. Whether you are analyzing gene expression in a collection of samples or transcription factor binding motifs in genomic atlases, you will need external information such as a reference genome or a specific gene annotation. For these types of data, there are three major providers: Ensembl (Yates et al. 2019), UCSC (Kent et al. 2002), and NCBI (Sayers et al. 2022), and many model-system specific providers, such as GENCODE (Frankish et al. 2021), ZFIN (Ruzicka et al. 2019), FlyBase (Thurmond et al. 2019), WormBase (Harris et al. 2019), Xenbase (Karimi et al. 2018), and more. Providers have different approaches to compiling genome assemblies and gene annotations, which affect formats, format compliance, naming, data quality, available versions, and release cycle. These differences significantly impact compatibility with research (Zhao and Zhang 2015), tools and (data based on) other genomic data.

You could try to find genomic data yourself, but there are many options. Ensembl, UCSC, and NCBI each have FTP archives, web portals, and REST APIs, which you can use to search their individual databases. Alternatively, there are several tools that can be used to access some of these databases programmatically, such as ncbi-genome-download (https://github.com/kblin/ncbi-genome-download) and ucsc-genomes-downloader (https://github.com/LucaCappelletti94/ucsc_genomes_downloader). However, none of these can search, compare, or download from all major genome providers. Furthermore, downloading and processing genomic data manually can be tedious, error-prone, and poorly reproducible. This can be remedied by a data management service or tool, such as iGenomes (https://support.illumina.com/sequencing/sequencing_software/igenome.html), refGenie (Stolarczyk et al. 2020), or Go Get Data (Cormier et al. 2021). These tools solve the issue of automation and reproducibility; however, they only provide genomic data for several commonly used organisms. Additionally, there is no programming interface to use the provided data.

We have developed genomepy to (i) find genomic data on major providers, (ii) inspect gene annotations, (iii) select the genomic data best suited to your analysis, and (iv) provide a suite of functions to peruse, manipulate, and use the data. Selected data can be downloaded from anywhere and is processed automatically. To ensure reproducibility, data sources and processing steps are documented and can be enhanced further by using a data manager. Genomic data can be loaded into genomepy, which uses and extends on packages including pyfaidx (Shirley et al. 2015), pandas (McKinney 2010), and MyGene.info (Xin et al. 2016) to rapidly work with gene and genome sequences and metadata. Similarly, genomepy has been incorporated into other packages, such as pybedtools (Dale et al. 2011) and CellOracle (Kamimoto et al. 2020). Genomepy can be used on the command line and through the (fully documented) Python API, for a one-time analysis or integration in pipelines and workflow managers.

## 2 Features of genomepy

The key features of genomepy are (i) providing an overview of available assemblies with the search function, (ii) downloading and processing of a selected assembly, with the install function and (iii) using reference assemblies and associated data through the Python API. A comprehensive overview of the features of genomepy compared to similar tools can be found in Supplementary Table S1.

**Figure 1:**
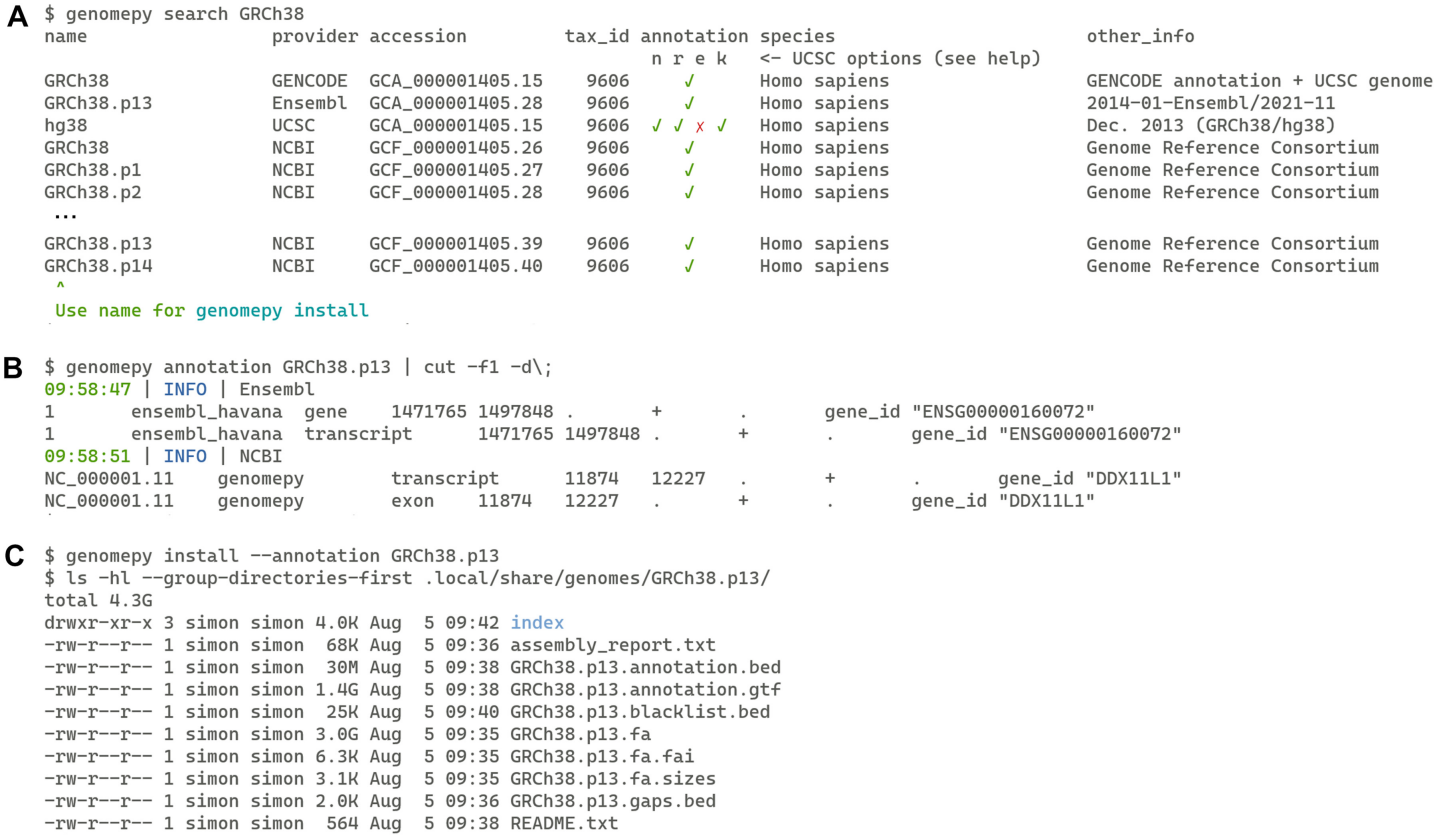
Examples of the genomepy command-line functions. (A) The search command searches providers for the given text, assembly accession or taxonomy identifier. (B) The annotation command provides a sample of the available gene annotation files. (C) The install command downloads the genome sequence, associated files and, optionally, gene annotation

The search function queries the databases of NCBI, Ensembl, UCSC, and GENCODE (caching the metadata), for text, taxonomy identifiers, or assembly accession identifiers. The input type is automatically recognized and used to find assemblies that have the text in the genome names or various description fields, matches the taxonomy identifier, or (partially) matches the assembly accession. The output of the search function is a table with rows of metadata for each assembly found. The metadata contains the assembly name and accession, taxonomy identifier, and indicates whether a gene annotation can be downloaded (or which of the four UCSC annotations) (see Fig. 1A). Snippets of available gene annotation(s) can be inspected with the annotation function (Fig. 1B).

An assembly name can be passed to the install function (Fig. 1C). The genome FASTA file is downloaded with the desired sequence masking level and alternate sequences (softmasked and none by default, respectively). Alternate sequences reflect biological diversity and are often contained in reference assemblies. During sequence alignment however, similar reference sequences result in multiple alignments, leading to loss of data [as discussed in Church et al. (2015)]. Additional filters may be passed to either include or exclude contigs (chromosomes, scaffolds, etc.) by name or regex pattern. Once processed, the genome is optionally compressed and a genome index is generated using pyfaidx, as well as contig sizes and contig gap sizes.

Gene annotations come in a variety of recognized formats (GFF3, GTF, and BED12). The install function will download the most descriptive format available, to output the commonly used GTF and BED12 formats. Contig names of the genome and gene annotation sometimes mismatch, which makes them incompatible with tools such as splice-aware aligners. Therefore, genomepy will attempt to match the contig names of the gene annotations to those used in the genome FASTA.

The install function can be extended with postprocessing steps via plugins. The options can be inspected and toggled with the plugin function. Briefly, the blacklist plugin downloads blacklists by the Kundaje lab (Amemiya et al. 2019) for the supported genomes. Other plugins support the generation of aligner indexes with separately installed tools, including DNA aligner indexes for Bowtie2 (Langmead and Salzberg 2012), BWA (Li and Durbin 2009), GMAP (Wu and Watanabe 2005), or Minimap2 (Li 2018), and splice-aware aligners such as STAR (Dobin et al. 2013) and HISAT2 (Kim et al. 2015).

Assemblies not present on the major providers can be processed similarly by supplying the URLs or file paths to the install function. For data provenance and reproducibility, a README file is generated during the installation process with time, source files, processing steps, and filtered contigs.

These features are available on both the command line interface and Python API. Additional features are available on the Python API, focused around two classes. The Genome class can be used to extract exact or random sequences from the FASTA, filter the FASTA and list the contigs, contig sizes, and contig gaps. The Annotation class can be used to browse and filter the BED12 or GTF files as pandas dataframes, map gene identifiers to other types using MyGene.info, map chromosome names to naming schemes of other major providers, and create a dictionary of any two GTF columns or attribute fields (to easily convert gene identifiers to gene names for instance).

## 3 Conclusion

Obtaining suitable reference genome data is a principal step in any genomics project. With genomepy, finding and downloading available assemblies becomes trivial. A genome, with the desired sequence masking, level of biological diversity, and contigs can be obtained with a single command.

Gene annotations in GTF and BED12 format, and matching the genome, can similarly be obtained, with further options available in the Python API. Whatever install options you choose are logged for reproducibility, allowing you to start your analysis with confidence.

## 4 Availability and implementation

The functionalities of genomepy are available from a command line interface, aimed at ease of use and integration in automated pipelines, and work efficiently with large genomic data as shown in Supplementary Table S2. Extended functionality is accessible using a Python application programming interface. The tool is freely available under the MIT license and can be installed using Bioconda (Grüning et al. 2018), pip (https://pypi.org), or directly used in workflows with our Docker (Merkel 2014) image or snakemake (Mölder et al. 2021) wrapper. The code is available on GitHub (https://github.com/vanheeringen-lab/genomepy) and archived on Zenodo (https://doi.org/10.5281/zenodo.1010458). Documentation is available on GitHub-pages (https://vanheeringen-lab.github.io/genomepy/).

## Supplementary Material

btad119_Supplementary_Data

## Data Availability

Not applicable.
