# Supplementary material for: genomepy: genes and genomes at your fingertips
Source: Bioinformatics. 2023 Mar 6;39(3):btad119. doi: 10.1093/bioinformatics/btad119 (PMC10017095; doi:10.1093/bioinformatics/btad119)
Supplement: btad119_Supplementary_Data [file btad119_supplementary_data.zip › 2023-01-20_Frolich_et_al.Table_S1.pdf]

	Interfaces				Compressed	Reproducible	Key features
	API	CLI	REST API	Website	data storage		
**genomepy**	☑	☑	☐	☐	☑	☑	Search, inspect, download & generate assembly data. Python API to use genomic sequences and to map gene and chromosome identifiers.
**ncbi-genome-download**	☑	☑	☐	☑	☑	☑	Mass download (subsets of) NCBI assembly data.
**ucsc-genomes-downloader**	☑	☐	☐	☐	☑	☑	Mass download (subsets of) UCSC genome assemblies.
**iGenomes**	☐	☐	☐	☑	☑	☑	Easily accessible preprocessed assembly data for (31) model organisms.
**Go Get Data**	☐	☑	☐	☐	☑	☑	Download & share scripts to reproduce various processed assembly data using the Conda infrastructure.
**Refgenie**	☑	☑	☑	☑	☑	☑	Share, download & generate assembly data via a private REST API server. Easily accessible preprocessed assembly data for (4) model organisms.

	Genome FASTA			Aligner indexes				Gene annotations				Other output
	Download	Share	Species	Download	Generate	Share	Aligners	Download	Share	Available	Output format	
**genomepy**	☑	☐	>106k	☐	☑	☐	6	☑	☐	~88k	BED & GTF	genome index, sizes, gaps & blacklist, NCBI assembly report
**ncbi-genome-download**	☑	☐	>105k	☐	☐	☐	0	☑	☐	~86k	GFF	various NCBI files & reports
**ucsc-genomes-downloader**	☑	☐	111	☐	☐	☐	0	☐	☐	0		
**iGenomes**	☑	☐	31	☑	☐	☐	3	☑	☐	108	refFlat & GTF	known abundant sequences
**Go Get Data**	☑	☑	5	☑	☐	☑	0	☑	☑	5	GTF	various filtered genome sequences, many genomic resources for human
**Refgenie**	☑	☑	4	☑	☑	☑	11	☑	☑	6	BED & GTF	genome blacklist, transcriptome & transcriptome index

Supplementary Table S1. Table of genomepy features as compared to similar tools.

Feature comparison of tools and services aimed at obtaining genomic reference assembly data.
