# Supplementary material for: genomepy: genes and genomes at your fingertips
Source: Bioinformatics. 2023 Mar 6;39(3):btad119. doi: 10.1093/bioinformatics/btad119 (PMC10017095; doi:10.1093/bioinformatics/btad119)
Supplement: btad119_Supplementary_Data [file btad119_supplementary_data.zip › 2023-01-20_Frolich_et_al.Table_S2.pdf]

Action	Max. RAM usage (MiB)	Time (sec)	Command
build cache	2812	61	genomepy search hg38
query cache	1863	10	genomepy search hg38
inspect annotations	87	10	genomepy annotation hg38 --provider UCSC
install genome	620	306	genomepy install hg38 --provider UCSC
install gene annotation	1729	42	genomepy install hg38 --provider UCSC --annotation
install blacklist	86	2	genomepy plugin enable blacklist; genomepy install hg38 --provider UCSC

Supplemental Table S2. Time and memory usage of core genomepy commands

Commands were executed up to 5 times and profiled using memory_profiler, averaging the maximum memory usage and total runtime.

Exact values vary depending on the selected assembly size, internet speed, provider latency and provider database size.
